# Concatenation of ‘alert’ and ‘identity’ segments in dingoes’ alarm calls

**DOI:** 10.1038/srep30556

**Published:** 2016-07-27

**Authors:** Eloïse C. Déaux, Andrew P. Allen, Jennifer A. Clarke, Isabelle Charrier

**Affiliations:** 1 Department of Biological Sciences, Macquarie University, Sydney, 2109, Australia; 2 Université Paris Saclay, Université Paris-Sud, CNRS, UMR 9197, Institut des Neurosciences Paris-Saclay, Orsay, 91405, France

## Abstract

Multicomponent signals can be formed by the uninterrupted concatenation of multiple call types. One such signal is found in dingoes, *Canis familiaris dingo*. This stereotyped, multicomponent ‘bark-howl’ vocalisation is formed by the concatenation of a noisy bark segment and a tonal howl segment. Both segments are structurally similar to bark and howl vocalisations produced independently in other contexts (e.g. intra- and inter-pack communication). Bark-howls are mainly uttered in response to human presence and were hypothesized to serve as alarm calls. We investigated the function of bark-howls and the respective roles of the bark and howl segments. We found that dingoes could discriminate between familiar and unfamiliar howl segments, after having only heard familiar howl vocalisations (i.e. different calls). We propose that howl segments could function as ‘identity signals’ and allow receivers to modulate their responses according to the caller’s characteristics. The bark segment increased receivers’ attention levels, providing support for earlier observational claims that barks have an ‘alerting’ function. Lastly, dingoes were more likely to display vigilance behaviours upon hearing bark-howl vocalisations, lending support to the alarm function hypothesis. Canid vocalisations, such as the dingo bark-howl, may provide a model system to investigate the selective pressures shaping complex communication systems.

According to Guilford and Dawkins^1^ selective pressures on signal design can act at the ‘content’ and/or the ‘efficacy’ levels. In other words, signals should both convey ‘information’ – as a predictable association between a signal’s feature and signaller’s characteristics (or event), which has evolved (or is maintained) because of its effect on receivers^2^ – and improve signal detectability, discriminability and memorability by receivers^1^,^3^. While historically researchers investigated signalling traits in isolation, it has since been recognized that animal signals often form a complex that includes several elements produced in the same or different modalities – termed ‘multicomponent’ or ‘multimodal’ signals respectively^4^,^5^. Several content and efficacy-based functional hypotheses as well as inter-signal interactions among different components, have been put forward to explain complex signals’ structures^5^. For instance, while some components may function to convey information, others may instead function to improve the overall detectability and/or discriminability of the signal e.g. ‘amplifier’ or ‘alerting and attention-altering’ hypotheses^5^. As an example, chickens, *Gallus gallus,* produce alarm calls that provide cues of predator class^6^ and aerial alarm calls include an initial ‘alerting’ component^7^, which likely improves the effectiveness of the signal by reducing receivers’ detection times and/or thresholds.

Multicomponent vocal signals that incorporate several components produced at intervals have been documented for many taxa including birds^6^, anurans^8^, whales^9^, bats^10^, primates^11^,^12^, rodents^13^ and rock hyraxes, *Procavia capensis*^14^. More rarely, multicomponent vocal signals can be formed by the uninterrupted concatenation of two or more elements. For instance, banded mongoose, *Mungo mungo*, produce contact calls that include a stable, individually distinctive component and a tonal component that varies according to the behaviour of the signaller^15^. Similarly, Campbell’s monkeys, *Cercopithecus campbelli campbelli*, can affix an invariable component to several vocalisations, and this affixation is dependent on the type of eliciting stimuli^16^.

Members of the Canidae family produce complex vocal signals about which we know surprisingly little^17^. These complex vocal signals have been described in detail for wolves, *Canis lupus*, and African wild dogs, *Lycaon pictus*^18^,^19^, although their functions have not been subjected to empirical investigation. Similarly, while dogs, *C. familiaris*, produce barks and growls as part of aggressive and playful vocal sequences, the effects of these vocalisations have been extensively studied independently (for reviews see refs 20 and 21), but not in combination.

Dingoes, *C. f. dingo*^22^ are medium-sized canids (likely descendant of early domesticated dogs) that arrived on mainland Australia 5 000 years ago^23^. Dingoes’ behavioural ecology closely resembles that of other wild canids as for example, they form packs of related individuals that cooperatively raise young, defend stable territories and display group hunting behaviours^24^,^25^,^26^. Their vocal repertoire is also similar to that of wolves’, being best described as a graded system of discrete vocalisations that can be produced independently or as part of multicomponent vocal sequences^27^.

Dingoes also utter a stereotyped, multicomponent ‘bark-howl’ vocalisation formed by the concatenation of a noisy, broadband bark segment and a low-frequency, tonal howl segment^27^ (Fig. 1). Both the bark and howl segments of dingo bark-howls can convey identity cues, although the level of individuality is higher in the howl segment^28^. Furthermore, the coding of identity cues (i.e. ‘individual signature characteristics’) appears conserved across howl segments of bark-howls and howl vocalisations^28^. Dingo howls (as opposed to howl segments) are produced singly or in bouts, often without any apparent external stimulus and allow for class-level recognition of familiar and unfamiliar conspecifics^29^ (Fig. 1). Bark vocalisations are typically produced as part of vocal sequences, which can include other vocalisations (e.g. woofs or growls), and can be elicited by both positive and negative external stimuli^27^. Unlike bark and howl vocalisations, bark-howls are highly context-specific, being produced in response to threats^27^,^30^. Observations of captive dingoes from different sanctuaries indicate that approach of an unfamiliar human (as well as the presence of a venomous snake) elicits bark-howling^27^. Evidence from wild dingoes across Australia corroborates this specificity, with all reports indicating that bark-howls are emitted upon dingoes detecting human observers^30^,^31^. Additionally, recordings of trapped animals show that the prevalence of bark-howl vocalisations is greatest post human arrival^32^, suggesting that bark-howling is not simply a stress and/or fear response but requires a specific external stimulus. As such, bark-howls have been hypothesized to serve as alarm calls, although empirical evidence is lacking^30^.

In this study, we aimed to investigate the function of bark-howls and the functional interactions between the bark and howl components. We designed a playback experiment in which we initially familiarized 18 adult dingoes to howl vocalisations from originally unknown exemplar individuals. Once dingoes had been familiarised (i.e. stopped responding) to the calls of their respective familiar exemplar, they were subjected to four types of stimulus series: bark-howl vocalisations from familiar exemplar individuals (BH), howl segments (truncated bark-howls) from familiar (FH) and unfamiliar (UH) exemplar individuals and bird calls (C), which served as control stimuli (Fig. 1). We did not test the effect of unfamiliar bark-howls on receivers because no evidence exists to suggest that these vocalisations are produced during inter-pack interactions and such a treatment would have limited biological relevance^30^. Similarly, we did not test the effect of bark segments alone to avoid having excessive treatment conditions and thus risking habituation/sensitization effects.

Instead, we selected these four conditions (BH, FH, UH treatments and a control) to test three hypotheses. Because howl segments share vocal identity signatures with howl vocalisations, we hypothesized that if this conserved individual identity information is adaptive then these cues should be salient to receivers. Specifically, we predicted that after familiarisation with an individual’s howl vocalisations, dingoes would discriminate between howl segments of bark-howls from a familiar and an unfamiliar exemplar, and display different responses to the two types of stimuli (prediction 1). Additionally, we hypothesized that bark segments would have an ‘alerting’ function and predicted that dingoes would display higher levels of attention when the bark segment was present compared to when only the howl segments of familiar individuals or control stimuli were played back (prediction 2). Lastly, we hypothesized that if bark-howls function as alarm signals, then receivers would display increased vigilance behaviours upon hearing full bark-howls compared to all other stimuli (prediction 3).

## Results

During the first two days of the experiment dingoes were presented with familiarization howl series consisting of 10 howl vocalisations from a given exemplar. Of the 18 dingoes considered in this investigation, 10 became familiarised to howl vocalisations of their allocated (originally unknown) exemplar individual by the end of the first day (criterion: no response to three consecutive howls) and the remaining eight became familiarised by the end of the second day. In subsequent days, each dingo was first presented with a familiarization howl series (to confirm familiarization) until the focal individual met the criterion, at which point we stopped the familiarization howl series and started the playback of one of the four test stimuli (BH, FH, UH or C). We performed three analyses to separately test each of the three predictions: 1) dingoes will display different responses to familiar and unfamiliar howl segments 2) will show increased attention level (i.e. look more toward the speaker) when bark segments are present and 3) will show increased vigilance behaviours (i.e. scan the environment) when barks are present.

### Effect of familiarity level on behavioural responses

Consistent with prediction 1, the final GLMM model, which tested whether dingoes could discriminate between familiar and unfamiliar howl segments, indicated that the overall strength of a dingo’s response (4-level ordinal scale) varied among stimulus types (log-likelihood ratio test or LRT: χ^2^ = 8.65, 3 d.f., P = 0.03; Table 1). Specifically, the strength of response to playbacks was greater for unfamiliar howl segments than for familiar howl segments (Z = 2.20, P = 0.03). This effect could not be attributed to the ‘novelty’ of the stimulus, as dingoes showed no difference in response between the familiar howl segment and control conditions (Z = 0.81, P = 0.42). We also found a sex effect (LRT: χ^2^ = 14.34, 1 d.f., P < 0.001; Table 1), with males showing stronger responses than females (Z = 3.59, P < 0.001). Test day (4 levels) did not have a significant effect (P = 0.10). Finally, differences among dingoes in the strengths of their responses appear negligible, as indicated by an estimated standard deviation near 0 for the magnitudes of random effects.

### Effect of stimulus type on attention level

Attention levels (i.e. proportion of calls within a test series that elicited a looking response) differed significantly among stimulus types (LRT: χ^2^ = 8.85, 3 d.f., P = 0.03; Table 1). Dingoes were more likely to continue looking toward the speaker during successive presentations of bark-howl calls compared to control sounds (Z = 2.51, P = 0.02) and familiar howl segments (Z = 2.51, P = 0.02), but not unfamiliar howl segments (Z = 1.11, P = 0.28). Thus, consistent with prediction 2, dingoes were more likely to continue reacting to successive vocalisations from familiar individuals, when the bark segment was included (Fig. 2). We also found a sex effect (LRT: χ^2^ = 9.15, 1 d.f., P = 0.002; Table 1), with males being more likely to display prolonged responses than females (Z = 2.96, P = 0.003), and a day effect (LRT: χ^2^ = 8.31, 3 d.f., P = 0.04). The magnitudes of random effects, as indexed by the standard deviation (1.11), were of similar magnitude to many of the model coefficients (Table 1), suggesting large differences among individuals in their overall attention levels.

### Effect of stimulus type on vigilance behaviours

The level of vigilance behaviour (i.e. whether dingoes assumed an erect posture, while looking at their surroundings) varied by stimulus type (LRT: χ^2^ = 11.75, 3 d.f., P = 0.008; Table 1). Consistent with prediction 3, dingoes were more likely to exhibit vigilance behaviours when bark-howls were broadcast compared to control sounds (Z = 2.87, P = 0.01), familiar howl segments (Z = 2.10, P = 0.04) and unfamiliar howl segments (Z = 2.08, P = 0.04). Thus, the presence of the bark segment caused dingoes to increase their vigilance (Fig. 3). We also found day effects (LRT: χ^2^ = 11.75, 3 d.f., P = 0.008; Table 1) but no sex effect (LRT: χ^2^ = 0.74, 1 d.f., P = 0.39; Table 1).

## Discussion

We found that dingoes displayed heightened responses to howl segments from unfamiliar conspecifics but not to howl segments from familiar exemplars. This pattern of responses is congruent with previous findings that reported an increase in responses to unfamiliar howl vocalisations in wolves^33^ and dingoes^29^ and demonstrates that dingoes can perform familiar-unfamiliar discrimination. Furthermore, dingoes were able to learn vocal cues from howl vocalisations and generalize these to new calls (i.e. howl segments). In other words, these results confirm that vocal identity signatures shared by howl vocalisations and howl segments^28^ are salient to receivers. Although it may be that howl segments have no/alternative functions, we propose that affixed howl segments may function as ‘identity signals’. Specifically, dingoes live in social groups composed of kin who presumably vary in their alarm calling thresholds (i.e. ‘reliability’). Thus, receivers would benefit from modulating their responses according to the reliability level of the calling individual, and vocal identity signatures may provide the means upon which this assessment is based. Furthermore, as pack members are related, kin selection could explain the affixation of the howl segment, as increased call individual distinctiveness would facilitate this process^34^. If howl segments serve as identity signals and function in individuality-based reliability assessment, then we would except dingoes to be capable of ‘true’ individual vocal recognition i.e. dingoes should have the ability to identify a conspecific (i.e. a specific pack member from another one) on the basis of his/her unique characteristics, from all other individuals in the pack^35^. This remains to be demonstrated in dingoes and other canids.

Additionally, dingoes are more likely to continue looking toward the speaker with successive presentations of familiar bark-howls compared to familiar howl segments and control sounds, supporting the hypothesis that the bark segment has an ‘alerting’ function. As dingoes did not show renewed attention in the control tests, we ruled out the possibility that this response was caused by the novelty of the stimulus. There was no difference in attention levels between familiar bark-howls and unfamiliar howl segments, although this was expected because both signals would provide cues of a potential threat. Indeed bark-howls may indicate the presence of a heterospecific threat and unfamiliar howl segments would be indicative of the presence of a conspecific intruder. That the bark segment has an ‘alerting’ effect supports earlier observational claims that barking in canids is associated with high levels of arousal, and functions to attract receivers’ attention^17^,^27^,^36^,^37^. The inclusion of a component, which increases conspecifics’ attention, may enhance the effectiveness of the signal through reducing the potential costs of delayed responses and/or missed signal detections. Evidence for such ‘alerting’ components exists for chicken and Richardson’s ground squirrel, *Spermophilus richardsonii* alarm calls^7^,^38^.

Sudden, plosive sounds with wide-spectrum characteristics have been proposed to evoke autonomic changes in attentional state^39^ and to maintain receivers’ attention^40^. Evidence exists in red deers, *Cervus elaphus*, where females were shown to exhibit increased attention to harsh roars (abrupt calls with a broadband spectrum) and to subsequent common roars (mostly harmonic structure) compared to common roars emitted earlier in the series^41^. Similarly, in yellow-bellied marmots, *Marmota flaviventris*, experimentally altered alarm calls in which a random noise section had been added, elicited longer vigilance behaviours than non-altered alarm calls or calls where silence had been inserted^42^. Importantly, these alarm calls do not naturally contain such non-linearities, indicating that it is the presence of the broadband section itself, and not its potential information content, which elicits increased attention. In other words, the presence of wide-spectrum components in a signal, such as red deers’ harsh roars and dingo bark segments, may predominantly be a reflexion of their efficacy effects on receivers. One important caveat is that bark segments could also convey adaptive information. Indeed, there is evidence to indicate that dogs’ barks can convey information on individual identity and the context of production^43^,^44^ and dingoes’ bark segments have similarly been found to be individually distinctive (although to a lesser extent than howl segments^28^). Thus, it could be that the redundant coding of identity cues in bark and howl segments facilitates individual discrimination^28^. Future research could aim to investigate the relative importance of the bark segment’s structure with regards to its potential information content and efficacy effects using sound resynthesis techniques.

Dingoes were also more likely to engage in vigilance behaviours upon hearing familiar bark-howls compared to all other treatments, supporting the hypothesis that bark-howls function as alarm signals. These results are congruent with findings of studies on alarm calling behaviours in other species^45^,^46^,^47^. Furthermore, the fact that the presence of the bark segment increased the probability of vigilance behaviours, suggests that this component is required for dingoes to recognize this signal as an alarm call. Future research should aim to investigate receivers’ responses to bark segments alone and in combination with howl segments of different conspecifics (e.g. unknown, ‘reliable’ and ‘unreliable’ callers) to determine whether barks have a general effect of increasing attention and vigilance behaviours and if the information conveyed in the howl segments may permit to modulate receivers’ behavioural responses. This would allow clarifying the extent of the interactions between the two components.

To the best of our knowledge, analogous experimental evidence of call concatenation altering receivers’ responses has only been shown in primate species, with propositions that combinatorial vocal sequences may represent precursor mechanisms to the more complex syntactic rules of human language^48^,^49^. Although call concatenation may function to alter the ‘semantics’ of the signal^16^, this study suggests that this phenomenon may also/alternatively be a reflection of selective pressures acting at the efficacy level. In other words, structurally different elements may be combined into single units not exclusively to modify ‘semantic’ content but because their efficacy effect(s) on receivers (e.g. eliciting autonomic changes in behaviours or reducing habituation) are adaptive in the communicative context. This phenomenon is already evident in the vocal sequences of many species^7^,^38^,^41^ and well acknowledged in the multimodal communication framework^5^.

Lastly, we found that males were overall more reactive than females, showing stronger responses (such as approaching the speaker) and higher attention levels. Whether this may reflect sex differences in defence behaviours as shown in other canids^50^ is unknown. We also found a test day effect on attention levels and vigilance behaviours. Graphical inspection of behavioural responses across days shows no linear trend, suggesting that this result is not attributable to habituation or sensitization effects during the experiment. Instead, this result may reflect differences among days (e.g. environmental conditions) and/or inter-individual variations in responsiveness.

Overall, our findings are consistent with the hypothesis that dingo bark-howls function as alarm calls. Future research should aim to further determine whether these calls may primarily function to warn conspecifics from a threat or to deter predators. These competing hypotheses could be tested based on the ‘audience effect’. Indeed, if bark-howls function to warn others of a danger, then dingoes would be more likely to call when in the presence of conspecifics. Conversely, if the call’s primary receiver is the ‘predator’ then dingoes would be expected to call regardless of the audience composition. Furthermore, our results suggest that the bark and howl segments have different roles. Specifically, the bark segment appears to serve as an ‘alerting’ component, and its function may predominantly relate to its efficacy effects. Howl segments convey identity cues that are sufficient for discrimination among individuals, and we propose that these components may function in identity-based reliability assessment. Dingoes’ bark-howls likely represent an excellent system to further investigate the interplay of content and efficacy pressures shaping signals’ designs and how this relates to signal structure, function and evolution.

## Materials and Methods

### Stimulus acquisition and preparation

**Figure 1:**
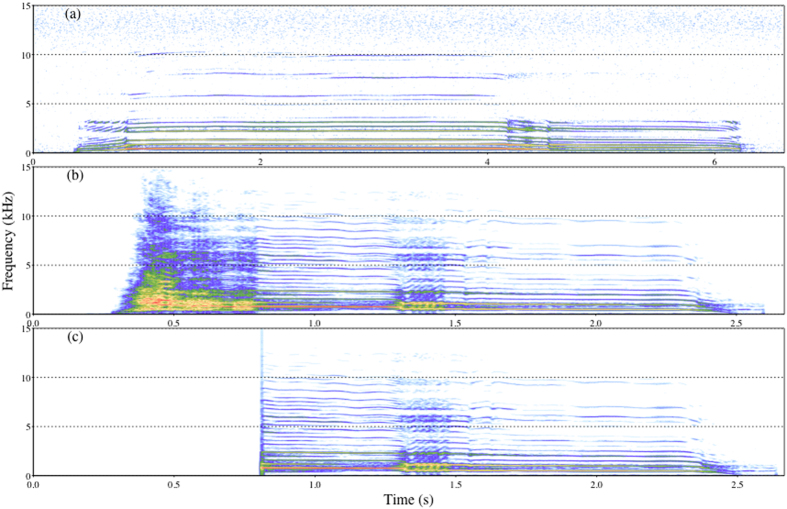
Example spectrograms of dingo howls and bark-howls. Spectrograms (FFT = 1024, Hanning window, 80% overlap) of (**a**) a howl vocalisation (**b**) a full bark-howl vocalisation (**c**) the howl segment of the bark-howl created by removing the bark segment.

**Figure 2:**
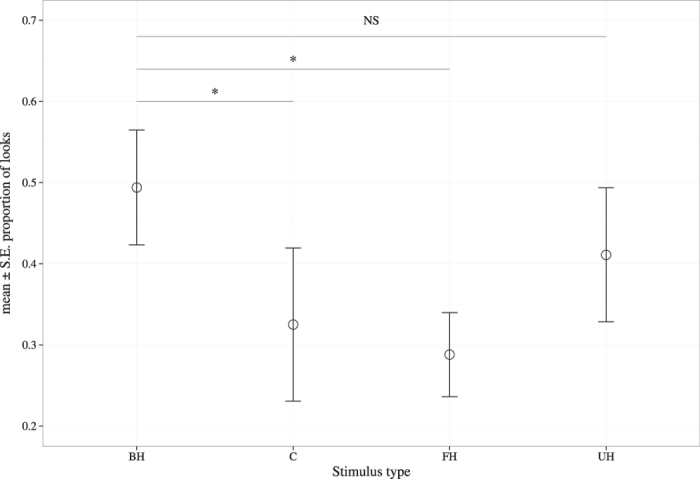
Dingoes’ attention levels according to stimulus type. Mean ± s.e.m. proportion of calls within a test series that elicited a looking response for each stimulus (BH = familiar bark-howl vocalisations, C = control sounds, FH = familiar howl segments, UH = unfamiliar howl segments). Asterisks indicate Hommel-adjusted, significant pairwise differences (at P < 0.05) between stimulus types coefficients of the fitted generalized linear mixed model (GLMM) as shown in Table 1.

**Figure 3:**
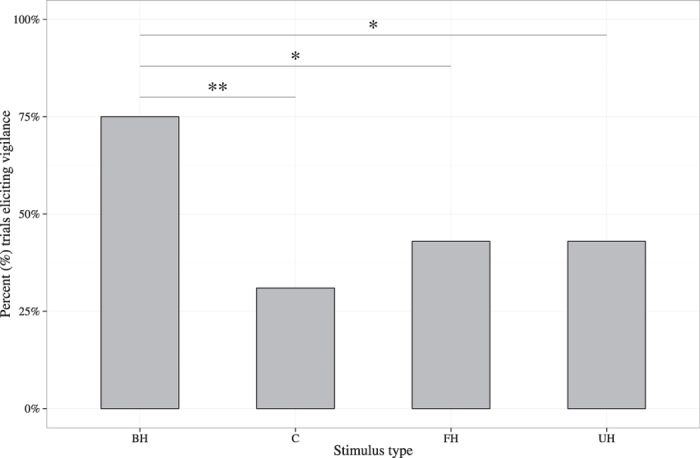
Dingoes’ vigilance behaviours according to stimulus type. Percentage of trials (n = 16 per treatment) that resulted in dingoes exhibiting vigilance behaviours for each stimulus type (BH = familiar bark-howl vocalisations, C = control sounds, FH = familiar howl segments, UH = unfamiliar howl segments). Asterisks indicate Hommel-adjusted, significant pairwise differences (with **P ≤ 0.01, *P < 0.05) between stimulus types coefficients of the fitted generalized linear mixed model (GLMM) as shown in Table 1.

Howl vocalisations and bark-howls were selected from a database of recordings collected since 2012 from captive dingoes housed in two sanctuaries: Colong Station and Secret Creek Reserve, New South Wales, described elsewhere^27^. A total of 141 calls from nine dingoes were selected. To prevent any potential confounding effect of differences in social relationships between exemplar and test dingoes, all exemplar dingoes were initially unknown to the test animals (they were maintained in sanctuaries different from the one where we conducted the tests). Instead, we experimentally created ‘familiar’ conspecifics by broadcasting their howl vocalisations (hereafter termed ‘familiarization howl series’) to the test dingoes during familiarization trials performed for two days before starting the test trials (Table 2). Test stimuli were created using bark-howls recordings from these experimentally created ‘familiar’ and ‘unfamiliar’ exemplars, such that we had three treatment types: 1) full bark-howl series from familiar exemplars (BH), 2) howl segment series from familiar exemplars (FH), and 3) howl segment series from unfamiliar exemplars (UH). To create howl segment series we truncated bark-howls, such that only the tonal segment was kept (Fig. 1). Control stimuli (C) were created using calls from six bird species occurring in the vicinity of the test sanctuary.

Familiarization howl series consisted of 10 different, randomly selected howl vocalisations from a given exemplar, separated by 3s silent intervals. The test (i.e. familiar bark-howls, familiar howl segments and unfamiliar howl segments) and control series consisted of five calls from a given exemplar (or a given bird species for the control stimuli), randomly selected and separated by 3s silent intervals. With the exception of one unfamiliar exemplar for whom we only had three vocalisations, all test series consisted of non-repeating calls. We applied a high-pass filter (100 Hz cut-off, 36 dB) with peak amplitude normalization (−2 dB) using Audacity 2.0.3^51^, to all series and a total of 104 unique stimulus tracks were thus created.

### Study site and animals

All tests were conducted at the Dingo Discovery and Research Centre, Toolern Vale, Victoria, during 17–24 September 2015, using 18 (10 females and 8 males) adult dingoes (2–10 years old). Dingoes were maintained in pairs in 2 × 5 m kennels with both indoor and outdoor compartments, containing elevated benches and covered beds. These dingoes were fed once daily a diet of dry dog food and/or meat carcasses and water was provided ad libitum. Typically, each pair is allowed daily access to one of four large outdoor paddocks. However, to avoid having individuals hearing playback stimuli outside of their allocated trials, during the experiment, dingoes were only allowed access to these enclosures when no testing was being conducted.

We took three additional measures to further reduce the chances of test individuals hearing the playback stimuli outside of their allocated trials. Firstly, tests were conducted in one of the four enclosures (690 m^2^), which was located ~80 m away and downhill from the holding kennels, such that with the exception of the focal individual, none were in close proximity. Secondly, the experimenter (E1) and equipment were located in an adjacent enclosure facing away from the kennels, as to avoid having the sound propagate towards the kennels where the other individuals were held. Finally, the Premio 8.2 speaker (T.A.G co.), positioned against the fence, behind a natural bush and connected to a MacBook Pro laptop was used to broadcast stimuli, at a natural but relatively low amplitude of 75 ± 5 dB SPL (measured at 1 m using a RadioShack 33–2055 digital sound level meter, peak amplitude, C-frequency and slow time weighting). The experimenter (E1), who operated the laptop, was in view of the dingoes but sat quietly and motionless on the ground, approximately 5 m away from the speaker. Dingoes’ responses were recorded using a Sony Handycam HDR-PJ760 camera mounted on a tripod.

### Experimental procedure

Each test dingo was randomly assigned to one familiar exemplar, one unfamiliar exemplar and one bird species, such that all received a unique combination of treatment and control series. Every individual received 6 trials in total, 2 initial familiarization trials to familiarize them to the howl vocalisations of their allocated familiar exemplar, and 4 consecutive test trials (3 treatments and 1 control trial). Dingoes were tested once daily, at the same time of day, with test trial order randomized and counter-balanced across subjects and test days.

All trials followed the same procedure, with the focal individual being removed from the holding kennels and brought to the test enclosure. Individuals were given at least 1min to explore the test enclosure. To control for dingoes’ attention immediately prior to stimulus presentation and ensure that they could hear the stimuli and were in view of the camera, we started the playbacks when the focal animal was occupied in sniffing one of four bushes that were within 10–15 m of the speaker. During familiarization trials we presented one full series of howls (i.e. 10 vocalisations) and recorded whether dingoes looked at the speaker for each call. We defined a ‘look’ as a marked head movement that stopped when the individual was looking straight at the hidden speaker. We arbitrarily set the criterion for familiarization as an absence of look toward the speaker for three consecutive howl presentations. Test trials were identical, with the exception that familiarization howl series were stopped as soon as the subject had reached the criterion (i.e. no look to three consecutive howls), and followed by a test stimulus series (i.e. either bark-howls, familiar howl segments, unfamiliar howl segments or control stimuli). This procedure allowed confirming dingoes’ familiarization to howl vocalisations and controlled for their attention immediately before test stimulus presentation. Trial durations were 10–30 min.

### Behavioural measures

Behavioural responses were scored from the videos using VideoPad 3.34 (NCH Software) and the experimenter E1 was blind to the treatment condition. We recorded three behavioural responses: 1) number of looks, 2) approach toward the speaker (i.e. positive phonotaxis) and 3) vigilance behaviours including ‘watching’ and ‘focused’. We recorded the number of looks for a given trial by scoring whether dingoes looked toward the speaker for each of the five calls of the test series. When the focal individual’s head was not clearly visible, we coded the response as missing for that call (7% of 360 observations). For each trial, we scored the presence/absence of approach behaviours, defined as a movement that decreased the distance between the focal individual and the speaker and exhibited during stimulus presentation. Lastly, we scored the presence of vigilance behaviours, if upon stimulus presentation, dingoes interrupted their behaviours and displayed either ‘watching’ or ‘focused’ behaviours^27^. ‘Watching’ dingoes maintained their heads above shoulder height, moving it in a scanning motion, while ‘focused’ dingoes kept their head immobile while looking at a specific feature of their surroundings (excluding the speaker). In both cases, the tail was typically hanging low and the individual was standing erect or moving at a walk/trot pace.

### Statistical analyses

A second observer (E2), who did not take part in the tests and was naive to the research question and experimental design, independently scored all 72 trials. For approach and vigilance responses, we assessed inter-observer reliability between E1 and E2 by calculating a concordance agreement as C = (A_o_ + A_no_)/(N), where A_o_ and A_no_ are the number of trials where both observers agreed on the presence and absence of the behaviour respectively, and N is the total number of trials (N = 72) and its associated Kappa statistic^52^. Kappa values lower than 0.4 are considered as poor, values ranging between 0.4 and 0.6 as fair agreement, those between 0.6 to 0.75 are good agreement, and values greater than 0.75 indicate excellent agreement^52^. We performed the same analysis for the number of looks response, except that we scored whether E1 and E2 agreed on the presence of a look for each of the five calls of a trial (excluding missing values N = 305 observations). Inter-observer reliability was good to excellent for all response variables, with a concordance of 84.7% (Kappa = 0.67) for approach behaviours, 79.2% (Kappa = 0.64) for vigilance behaviours and 79.3% (Kappa = 0.81) for the number of looks.

Differences in behavioural responses among treatments were analysed using generalized linear mixed models (GLMMs), fitted by maximum likelihood with Laplace approximation. To generate parsimonious models, we used a two-step procedure. We first used AIC to determine a parsimonious random-effects structure based on a full model (i.e. a model containing all candidate fixed-effect terms), and then removed statistically insignificant fixed-effect terms^53^. Fixed effects included in the full models were stimulus type, dingo sex, and test day. For the first step, we compared models with intercepts that varied randomly by dingo to models that varied randomly for unique combinations of dingo and stimulus. For the second step, variables were pruned using backward stepwise selection based on likelihood ratio tests^53^ and distributions of random coefficients were visually inspected for normality. To test for significant differences between stimulus types, we performed multiple pairwise comparisons on a priori contrasts, using the Hommel correction^54^. All tests were two-tailed and α ≤ 0.05. All statistical analyses were conducted in R^55^ using the lme4^56^ and multcomp^57^ packages.

To assess if dingoes’ responses varied according to the familiarity of howl segments (prediction 1) we used a composite ordinal scale, successfully used in previous research on dingoes’ discrimination abilities^29^. Responses were classified on a four-level scale with ‘low’, ‘medium’ and ‘high’ scores given when dingoes looked toward the speaker on 0–1, 2–3 and 4–5 calls without approach, while a response was scored as ‘strongest’ if it included an approach toward the speaker. We used a Poisson model (log link) and performed pairwise comparisons between the familiar howl segment and unfamiliar howl segment conditions, and between the familiar howl segment and control treatments.

Dingoes’ responses to acoustic stimuli are typically short-lived and they naturally stop attending to stimuli after a few consecutive presentations^29^. Thus, we used the number of looks elicited as an index of attention level to test prediction 2. We tested for differences in attention among treatments using a binomial model (logit link) with the number of successes (i.e. looks) and failures (i.e. no look) within a trial as the response variable. Responses to bark-howl stimuli were compared to all other treatments.

Lastly, we tested for differences in dingoes’ likelihood to exhibit vigilance behaviours (i.e. whether dingoes interrupted their behaviours and scanned/watched their surroundings) among treatments (prediction 3) using a binomial model (logit link), excluding two individuals from the analysis, as they displayed no vigilance behaviour in any of the test trials. Pairwise comparisons were used to assess whether vigilance levels differed between the bark-howl treatment and all other conditions.

### Ethics statement

This research was conducted in accordance with the Australian Code of Practice for the Care and Use of Animals for Scientific Purposes (NHMRC, 2013). All procedures were approved under Macquarie University Animal Ethics Committee protocol number 2011/031 and 2012/053.

## Additional Information

**How to cite this article**: Déaux, E. C. *et al*. Concatenation of ‘alert’ and ‘identity’ segments in dingoes’ alarm calls. *Sci. Rep.* **6**, 30556; doi: 10.1038/srep30556 (2016).

## Figures and Tables

**Table 1:** Results of GLMM models testing the effects of sex, test day (1 to 4) and stimulus type (BH = bark-howl vocalisations, FH = familiar howl segments, UH = unfamiliar howl segments, C = control sounds) on dingo behavioural responses, including likelihood ratio tests of fixed effects (P < 0.05 in bold), and summary statistics of all effects included in the final models.

Response	Predictor	Fixed Effect					Random Effect
		χ^2^	P	Level	Coef	s.e.	s.d.
Behavioural Response strength	Intercept (FH/Female)				−1.18	0.37	
(n = 18 dingoes)	Stimulus type	8.65	0.03	BH	0.79	0.38	
				C	0.34	0.41	
				UH	0.83	0.38	
	Sex	14.34	<0.001	Male	0.97	0.27	
	Day	6.17	0.1				
	Dingo						0
Attention	Intercept (BH/Female/Day)				−0.79	0.54	
(n = 18 dingoes)	Stimulus type	8.85	0.03	C	−1.41	0.56	
				FH	−1.37	0.55	
				UH	−0.58	0.53	
	Sex	9.15	0.002	Male	1.17	0.39	
	Day	8.31	0.04	Day2	0.65	0.55	
				Day3	1.16	0.56	
				Day4	−0.3	0.56	
	Stimulus:Dingo						1.11
Vigilance	Intercept (BH/Day1)				0.08	0.8	
(n = 16 dingoes)	Stimulus type	11.75	0.008	C	−3.07	1.07	
				FH	−2.02	0.96	
				UH	−1.94	0.93	
	Sex	0.74	0.39				
	Day	11.75	0.008	Day2	2.81	1.04	
				Day3	2.41	1.02	
				Day4	1.09	0.92	
	Dingo						0.61

**Table 2:** Break-down of the number of calls selected for each exemplar dingo and each category of stimulus (Howl = howl vocalisation, BH = full Bark-howl, FH = familiar howl segment, UH = Unfamiliar howl segment).

Dingo	Howl	BH	FH	UH
Bash*	14	9	9	—
Digger*	15	10	10	—
Jaroo*	17	10	10	—
Snooks*	23	8	8	—
AR	—	—	—	5
Bear	—	—	—	10
Bindi	—	—	—	3
Mootik	—	—	—	9
Rusty	—	—	—	8
Total	69	37	37	35

‘Familiar’ exemplars are identified by an asterisk.
